# Critical Role of Methylglyoxal and AGE in Mycobacteria-Induced Macrophage Apoptosis and Activation

**DOI:** 10.1371/journal.pone.0000029

**Published:** 2006-12-20

**Authors:** Helmy Rachman, Nayoung Kim, Timo Ulrichs, Sven Baumann, Lydia Pradl, Ali Nasser Eddine, Matthias Bild, Marion Rother, Ralf-Jürgen Kuban, Jong Seok Lee, Robert Hurwitz, Volker Brinkmann, George A. Kosmiadi, Stefan H.E. Kaufmann

**Affiliations:** 1 Max Planck Institute for Infection Biology, Department of Immunology Berlin, Germany; 2 Max Planck Institute for Infection Biology, Department of Molecular Biology Berlin, Germany; 3 Laboratory of Functional Genomics, Charité, Universitätsmedizin Berlin Berlin, Germany; 4 Max Planck Institute for Infection Biology, Core Facility Berlin, Germany; 5 Central Tuberculosis Research Institute, Department of Immunology 2 Moscow, Russian Federation; New York University School of Medicine, United States of America

## Abstract

Apoptosis and activation of macrophages play an important role in the host response to mycobacterial infection involving TNF-α as a critical autocrine mediator. The underlying mechanisms are still ill-defined. Here, we demonstrate elevated levels of methylglyoxal (MG), a small and reactive molecule that is usually a physiological product of various metabolic pathways, and advanced glycation end products (AGE) during mycobacterial infection of macrophages, leading to apoptosis and activation of macrophages. Moreover, we demonstrate abundant AGE in pulmonary lesions of tuberculosis (TB) patients. Global gene expression profiling of MG-treated macrophages revealed a diverse spectrum of functions induced by MG, including apoptosis and immune response. Our results not only provide first evidence for the involvement of MG and AGE in TB, but also form a basis for novel intervention strategies against infectious diseases in which MG and AGE play critical roles.

## Introduction

Tuberculosis (TB) represents a major threat to humankind, causing approximately 2 million deaths each year. In the majority of individuals infected with *Mycobacterium tuberculosis* (MTB), the bacilli cause long-term asymptomatic infection termed latent TB during which bacilli persist within granulomas in a dormant stage. Latently infected individuals have a ca. 10% risk of progression to clinical disease during their lifetime [1], [2].

Within granulomas, macrophages undergo apoptosis, which participates in host defence against TB [3], [4]. Correlation between apoptosis and virulence/persistence of mycobacteria remains controversial. Virulent MTB have been described to induce more profound apoptosis in macrophages and dendritic cells *in vitro* than attenuated *Mycobacterium bovis* BCG (BCG) [5]. Yet, evidence has also been presented that MTB attenuates host cell apoptosis in infected macrophages via induction of antiapoptotic mechanisms and/or upregulation of antiapoptotic genes [6]–[8]. Thus, although the exact impact of MTB on apoptosis remains unclear, a role of apoptosis in protection against, and pathogenesis of, TB is likely.

Apoptosis can be induced by various physical and chemical insults. Recent studies revealed that methylglyoxal (MG) induces apoptosis in a cell-type-dependent manner [9]–[11]. The cytotoxicity of MG involves apoptotic events. MG induces apoptosis in Jurkat cells by activating the c-Jun N terminal kinase (JNK) signal transduction pathway, which decreases the mitochondrial membrane potential, followed by caspase-3 activation [11]. In cultured HUVEC cells, MG induces tyrosine phosphorylation and aggregation of a number of cellular proteins [12]. In aortic VSMC from hypertensive rats, elevated levels of MG and advanced glycation end products (AGE) are found, in parallel with evidence that MG increases oxidative stress, activates NF-κB, and enhances ICAM-1 expression [13]. MG can modify proteins rapidly and cause cross-linking, probably changing the properties of these proteins and thus triggering intracellular responses. Abordo and Thornalley demonstrated that MG-modified human serum albumin (MG-HSA) induces increased expression of TNF-α in human monocytic THP-1 cells [14]. Together, these studies suggest that MG-modified proteins play an intriguing role in the development of distinct disease states.

Results of global gene expression analysis of MTB isolated from active pulmonary TB indicate that the tissue environment of MTB *in vivo*, particularly in granulomatous lesions, is rich in MG [15]. Since a significant number of macrophages in these lesions undergo apoptosis [3], we hypothesized that MG levels in macrophages determine apoptotic events during mycobacterial infection. To contest this hypothesis, we measured MG levels in macrophages during mycobacterial infection, characterized MG-induced apoptosis, and ascertained the relationship between elevated levels of MG/AGE and macrophage functions during mycobacterial infection. Our findings point to a close interrelation between MG/AGE and apoptosis as well as activation of macrophages during TB.

## Results and Discussion

### Mycobacterial Infection of Macrophages Triggers Increased MG Production

Our DNA array results suggest that the environment of tubercle bacilli at sites of infection is rich in aldehydes, especially MG [15]. To verify the assumption of an MG-rich environment during mycobacterial infection of the lung, we infected MH-S cells *ex vivo* with mycobacteria and measured relative MG levels in these cells. MH-S cells are an established murine alveolar macrophage-derived cell line. Alveolar macrophages play a pivotal role at the initial innate immune response against infection of tubercle bacilli via the respiratory tract [1]. Moreover, recent evidence reveals that alveolar macrophages are able to mount appropriate immune response [16]. We observed that MG levels significantly increased in MH-S cells 1 day after mycobacterial infection as determined by HPLC (Figures S1A–C). In three independent experiments, MG levels were 2.0–3.7 times higher in mycobacteria-infected macrophages as compared to noninfected macrophages. This level of augmentation is comparable to that in *in vitro*-induced hyperglycemia and in the blood of diabetic patients [17], [18].

These findings demonstrate for the first time that MG levels are significantly increased during mycobacterial infection of macrophages. In general, MG is a physiological product of various metabolic pathways, including anaerobic glycolysis, oxidative decomposition of polyunsaturated fatty acids as well as fragmentation of triosephosphate or catabolism of ketone bodies and threonine. MG, 3-deoxyglucosone (3-DG), and glyoxal (GO) are formed during each step of the glycation process by degradation of glucose or Schiff's base in early glycation, or from Amadori products such as fructosamine in the intermediate stages of glycation [19], [20]. MG levels in cells are regulated by several factors including the glyoxalase system and other components which influence the glycolysis pathway. The glyoxalase enzyme system in eukaryotic cells, which comprises glyoxalase I and II, is primarily responsible for detoxification of MG [21]–[23]. Increased levels of glucose or deprivation of phosphate in the cytoplasm can induce MG production by interfering with the glycolysis pathway [24]. It is thus possible that mycobacterial infection of macrophages triggers shifts in these metabolic pathways leading to increased MG production. We cannot formally exclude that mycobacteria directly contribute to increased MG levels of infected macrophages. For instance, reactive oxygen radicals produced during mycobacterial infection may oxidize polyunsaturated fatty acids, which represent abundant components of the mycobacterial cell wall, leading to their decomposition and MG formation. Moreover, our microarray data from pulmonary TB patients indicate that MTB experiences nutrient deprivation at sites of pulmonary TB [15]. Such conditions may alter MTB metabolic pathways leading to MG formation. Due to its highly reactive and small molecular nature, MG can easily diffuse out of mycobacterial cells into host cells.

### MG Induces Apoptosis in MH-S Cells

In an attempt to unravel the function of MG during mycobacterial infection, we assessed whether MG plays a role in apoptosis of infected MH-S cells. Basically two observations support this notion. First, MG induces apoptosis in different cell lines [11]; second, granuloma formation is accompanied by apoptosis of macrophages [3]. Consistent with this, a high proportion of apoptotic cells was found in bronchoalveolar lavage from patients with reactive or disseminated TB [25]. *In vitro* models have demonstrated that mycobacteria cause apoptosis and apoptosis of host macrophages has been claimed to restrict growth of intracellular mycobacteria [6].

**Figure 1:**
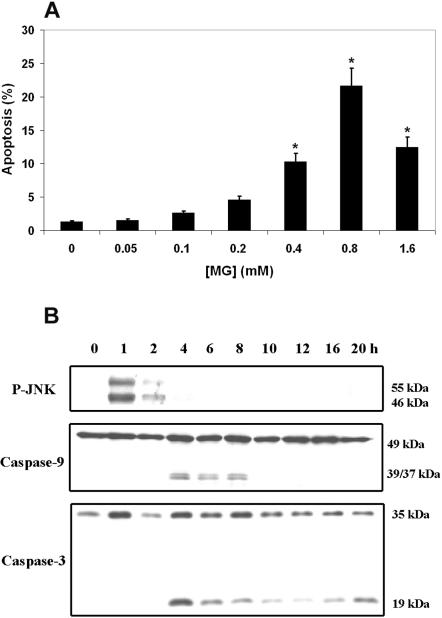
MG Induces Apoptosis in the Murine Alveolar Macrophage Cell Line MH-S (A) MH-S cells were treated with different concentrations of MG for 24 h. Cells were stained with propidium iodide and analyzed using a FACS instrument. Apoptosis was scored as the percentage of cells appearing in the area below the G1/G0 peak, representing cells containing DNA<2N. Error bars represent±SD. Statistical significance was determined by Student's t test; *, *P*<0.001 for MH-S cells treated with 0.4, 0.8, and 1.6 mM MG versus untreated MH-S cells. (B) Western blot analysis was used to detect the activation of components of apoptotic signals, JNK, caspase-3, and caspase-9. JNK was activated as early as 1 h after MG treatment, followed by the activation of caspase-3 and caspase-9.

Accordingly, we first investigated the ability of MG to induce apoptosis in MH-S cells. Apoptosis occurred in this cell line, as assessed by staining of chromosomal DNA with propidium iodide (PI) followed by cellular analysis using the FACScalibur flow cytometer (Figures 1A and S2). The percentage of apoptotic cells peaked at an MG concentration of 0.8 mM. At an MG concentration of 1.6 mM the proportion of apoptotic cells decreased. A possible explanation for this observation is that MG induces profound necrosis rather than apoptosis in MH-S cells at this high concentration, as observed recently in Jurkat cells [11]. Alternatively, at this high concentration MG triggers growth arrest at the G1 phase in MH-S cells as reported previously for other cell types [10]. We observed increased externalization of phosphatidylserine, as an early sign of apoptosis 4 h after MG treatment (Figure S3) indicating that apoptotic signals were induced prior to this time point.

JNK plays an important role in apoptotic processes [26]–[28]. The induction of apoptosis by MG has been correlated with the activation of c-Jun N-terminal kinase (JNK) [11]. To examine the apoptotic route of MG-treated MH-S cells, total proteins of MG-treated MH-S cells were isolated at different time points after treatment and JNK, caspase-3, and caspase-9 activation were determined. JNK was activated as early as 1 h after MG treatment and this activation was maintained for 2 h. Caspase-3 and caspase-9 activation occurred 4 h after MG treatment (Figure 1B).

The activation of JNK can occur upstream or downstream of caspase activation. When JNK activation precedes caspase activation, it is usually required for apoptosis. This is particularly relevant when cells are exposed to insults caused by various chemical agents, irradiation, or oxidation [29], [30]. Our results suggest that activation of JNK precedes the onset of apoptosis, and initiates an intracellular signaling cascade in preparation for apoptosis induced by MG in MH-S cells. We conclude that JNK activation is required for MG-induced apoptosis of MH-S cells. Our results agree with previous reports [11], extending the apoptosis-inducing potential of this small molecule to alveolar macrophages.

### Increased AGE Levels in Mycobacteria-infected Macrophages

**Figure 2:**
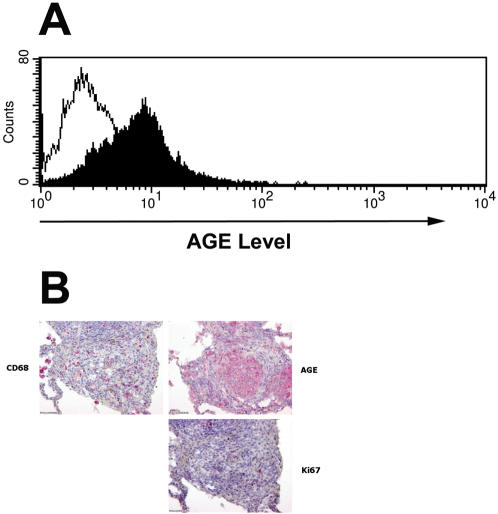
Mycobacterial Infection of Macrophages Stimulates AGE Formation. (A) Macrophages were infected with BCG for 1 day and AGE levels were determined by FACS analysis. AGE formation was strongly increased during infection of macrophages. (B) Significant formation of AGE was also observed at sites of active TB; upper left: staining with mAb against macrophage marker (CD68), upper right: staining with mAb against AGE, lower right: staining with mAb against marker for activated macrophages (Ki-67). Consistent results were observed for three TB patients.

Under physiological conditions, most MG is bound to cellular proteins as adducts formed with Lys, Arg, and Cys residues. Although the reaction with Cys is considered reversible, elevated MG concentrations can irreversibly modify Lys and Arg residues through AGE formation. AGE can enhance formation of oxygen radicals with subsequent activation of NF-κB and release of proinflammatory cytokines [31], [32]. AGE can stimulate mitogen-activating protein kinase (MAPK), activator protein-1 (AP-1), and induce expression of transforming growth β1 (TGF-β1) and of extracellular matrix (ECM) proteins [33], [34]. It is, therefore, conceivable that abundant MG levels present during mycobacterial infection of macrophages lead to AGE formation of distinct proteins, which then trigger changes in global gene expression. In hyperglycemia, increased levels of MG are accompanied by elevated AGE formation [17]. We observed abundant AGE during mycobacterial infection of macrophages (Figure 2A). More importantly, we detected AGE in TB lesions of patients. Figure 2B shows a histological specimen of lung lesion from a TB patient. AGE staining colocalizes with the accumulation of macrophages suggesting that macrophages are one of the main sources of AGE in active TB. Thus, formation and accumulation of AGE occurs in TB. Until recently, increased AGE formation has been mostly associated with diabetes, renal failure, aging, and cancer [35]. Therefore, our observation extends the roles of AGE to chronic infectious diseases such as TB and hence, provides new insights into regional immunity against, and pathogenesis of, a chronic bacterial infection.

### Mycobacteria-induced Apoptosis Requires MG and AGE

**Figure 3:**
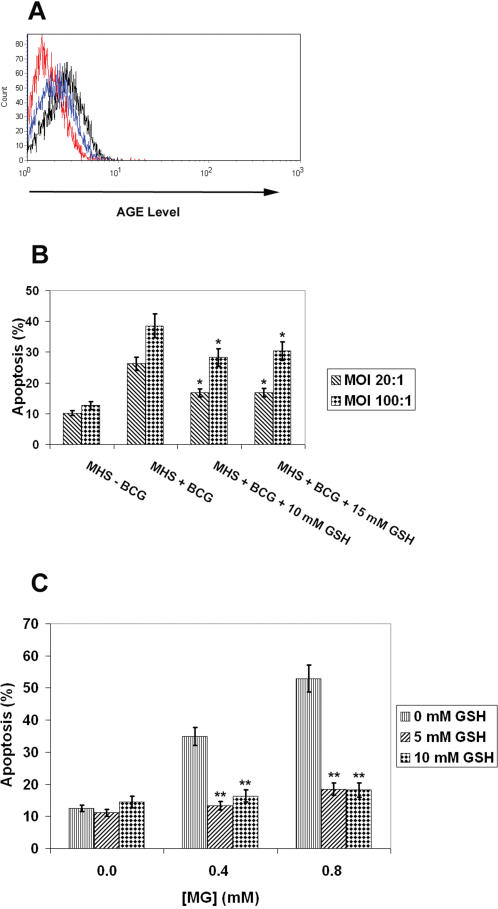
GSH Reverses Apoptotic Effects of MG/AGE on Macrophages. (A) Macrophages were pretreated with GSH and infected with mycobacteria for 1 day. As a positive control, macrophages were infected with mycobacteria without pretreatment with GSH. AGE levels were determined using a FACS instrument. Red line: negative control (without infection), black line: positive control, blue line: pretreatment with GSH. The augmentation of AGE levels was reduced during mycobacterial infection when macrophages were pretreated with GSH. (B) Pretreatment of macrophages with GSH reduced the proportion of apoptotic cells during mycobacterial infection. Error bars represent±SD. Statistical significance was determined by Student's t test; *, *P*<0.05 for MH-S cells pretreated with GSH and infected with mycobacteria versus MH-S cells infected with mycobacteria without pre-treatment with GSH. (C) Pretreatment of macrophages with GSH reduced the proportion of apoptotic cells during MG treatment. Error bars represent±SD. Statistical significance was determined by Student's t test; **, *P*<0.001 for MH-S cells pretreated with GSH and treated with MG versus MH-S cells treated with MG without pretreatment with GSH.

Reduced glutathione (GSH) serves as a cofactor of the glyoxalase system and catalyzes the conversion of MG to D-lactate. MG binds nonenzymatically to GSH forming hemithioacetal, which in turn serves as a substrate for glyoxalase I (GLO1). Glyoxalase II (GLO2) hydrolyzes the product from the GLO1-catalyzed reaction, S-D-lactoylglutathione to D-lactate. GSH is also a well-known antioxidant, which neutralizes oxidative radicals, including oxygen radicals. Under various experimental conditions, induction of apoptosis is preceded by a rise in intracellular oxygen radicals [9]. MG can induce generation of reactive oxygen radicals through multiple pathways [36]–[38] and these radicals contribute to apoptotic events induced by MG [39]. We show that preincubation of macrophages with GSH reduced AGE formation after infection with mycobacteria (Figure 3A). At the same time we observed a significant reduction in apoptosis (Figure 3B). Similar results were obtained for MG-treated macrophages, which had been preincubated with GSH (Figure 3C). This finding suggests that mycobacteria-induced apoptosis of macrophages involves elevated AGE levels.

### MG Modulates Expression of Apoptosis- and Immune Response-related Genes

**Figure 4:**
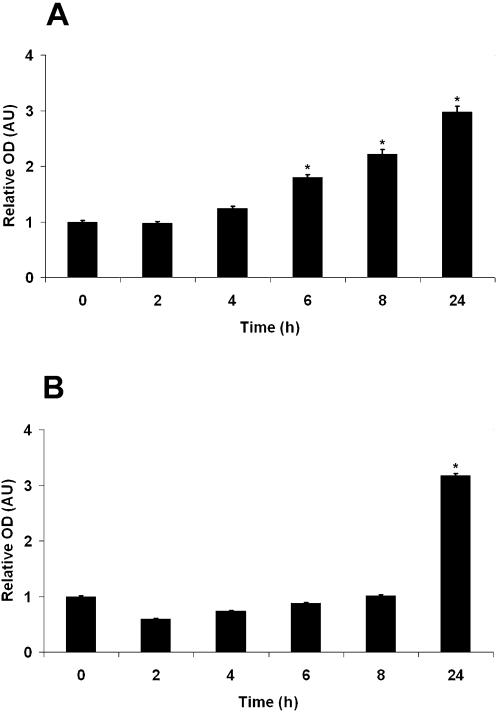
Cytokine and Chemokine Induction by MG. MH-S cells were treated with 0.8 mM MG. TNF-α and CXCL10 concentrations in the culture medium were measured at various time points as indicated on the X axis. Relative OD values were calculated by setting the OD value to 1 at time 0 h. A significant increase of TNF-α (A) and CXCL10 secretion (B) was observed after MG treatment of MH-S cells. This confirms the results of microarray experiments. Error bars represent±SD. Statistical significance was determined by Student's t test; *, *P*<0.05 for MH-S cells treated with MG (6, 8, and 24 h after treatment for TNF-α and 24 h after treatment for CXCL10) versus untreated MH-S cells.

In an effort to unravel the mechanism(s) of apoptosis induced by MG, we performed global gene expression profiling of MG-treated macrophages. Promptly after MG treatments (30 min), a cluster of genes involved in apoptosis were induced (Tables S1–6) and many of these were increasingly expressed at later time points. TNF-α was induced at all time points analyzed confirming previous reports that MG and AGE can induce TNF-α production [14]. Increased transcription of TNF-α was accompanied by a high level of TNF-α protein in supernatants of macrophages treated with MG (Figure 4A). In addition, several genes associated with TNF-α, such as TRAF1, TRAF2, and members of the TNF-α receptor superfamily were also induced (Tables S1–6). The expression of some DNA damage-inducible genes, such as DDIT3 and GADD45G was strongly elevated, indicating that MG directly or/and indirectly causes DNA damage as a characteristic feature of apoptosis (Tables S1–6).

Notably, MG does not only induce apoptosis-related genes, but also a large number of genes related to macrophage activation and induction of immunity. Beside TNF-α, a series of genes belonging to the chemokine and cytokine families (ligands and receptors) were induced (Tables S1–6). Among highly induced chemokines were CXCL2 and CXCL10, which are highly abundant in lung granulomas induced by mycobacteria [40]. The high expression of CXL10 was confirmed by real-time RT-PCR (Table S7) and ELISA (Figure 4B). Furthermore, real-time RT-PCR results showed that pretreatment of macrophages with GSH reduced the induction of the corresponding genes (Table S7). This directly indicates the involvement of MG in induction of these genes.

Lung granulomas represent the hallmark of pulmonary TB and the prime focus for the continuous crosstalk between MTB and immune cells [41]. Induction of immunity involves elaborate interactions between chemokines and cytokines produced by immune cells as well as local tissue cells [40], [41]. TNF is a pivotal player in granuloma formation [42], [43]. TNF-deficient mice infected with MTB are highly susceptible to disease, and granuloma formation is severely impaired [42]. Therapeutic TNF neutralization in rheumatoid arthritis patients with latent MTB infection is associated with increased risk of reactivation TB [44]. Thus, considering the large number of genes related to chemokines and cytokines, the expression of which is strongly regulated by MG, we suggest that MG and AGE play pivotal roles in immunity to TB with a focus on the granulomatous lesion. This assumption is supported by the fact that >70% of the genes, which were regulated by MG (this study), are similarly regulated in granulomas of MTB-infected mice (Tracy Walker, personal communication).

Our global gene expression profiling of MG-treated macrophages revealed that MG effects the expression of numerous genes, many related to apoptosis, activation, and immunity. Moreover, MG seems to effect genes associated with regulation of cellular growth. The glyoxalase system and MG have been proposed as major regulators of cell division based on their ubiquitous occurrence and the high reactivity of MG with cellular thiols underlying cell division [45], [46]. In sum, our experiments revealed numerous roles of the small molecule MG in diverse cellular events. Understanding the mechanism(s) involved in these events can provide the basis for designing novel intervention strategies against infectious diseases in which MG and AGE are critical.

## Materials and Methods

### *In vitro* Culture of Bacteria and Culture Conditions

BCG Copenhagen organisms were grown in Middlebrook 7H9 media supplemented with 10% (v/v) ADC (Difco Laboratories) and 0.05% (v/v) Tween 80 (Sigma) to mid-log phase at 37°C with shaking at 90 rpm in screw-capped bottles.

### Culture of MH-S Cells

MH-S cells (ATCC No. CRL-2019) were cultured in RPMI medium supplemented with 10% FCS, 1 mM Na-pyruvate, and 2 mM L-glutamate, 10 mM HEPES, 0.05 mM 2-mercaptoethanol, 100 U/ml penicillin, and 10 mg/ml streptomycin sulphate. The cells were maintained in a humidified atmosphere of 5% CO_2_ at 37°C. During mycobacterial infection, culture medium without antibiotics was used. Infection of MH-S cells with BCG Copenhagen was performed as described previously [6], [7].

### Flow Cytometric Analysis of DNA Fragmentation

MH-S cells were seeded at 2.5×10^5^ cells/ml into a 24-well tissue culture plate for 1 day (2.5×10^5^ cells/well). After treatment with 0.8 mM MG (Sigma) for 24 h, cells were harvested, resuspended in 500 µl of propidium iodide buffer (0.1% Triton X-100, 1×PBS) containing 50 µg/ml propidium iodide (Sigma), and incubated for 15 min on ice. DNA fragmentation analysis was carried out using a FACScalibur flow cytometer (Becton Dickinson). Apoptosis was scored by the percentage of cells appearing in the area below the G1/G0 peak representing cells containing DNA<2N.

### Annexin V Binding

One million MH-S cells were seeded into a 6-well plate for 1 day and then treated with 0.8 mM MG, and harvested after MG treatment for 4, 6, and 24 h. The MH-S cells were harvested, washed twice with 1×PBS, resuspended in annexin V binding buffer, and stained with annexin V according to the manufacturer's instructions (Sigma) before flow cytometric analysis.

### Determination of MG Levels in MH-S Cells

Measurement of MG levels was performed as described previously [23] with a few modifications. MH-S cells (2×10^7^) were infected with BCG at MOI of 50∶1. One day after infection, MH-S cells were harvested, washed twice with 1×PBS, and lysed by sonication in 5 ml 1×PBS. Perchloric acid (PCA) (Sigma) and o-phenylenediamine (o-PD; Sigma) were added to a final concentration of 0.5 M and 5 mM, respectively. The mixture was incubated at 20°C for 24 h. PCA precipitate was removed by centrifugation. The supernatant was passed through a C_18_ solid-phase extraction cartridge (Waters Sep-Pak tC18 plus cartridge, Millipore), which had been prepared by flushing with 6–8 ml of acetonitrile and 6–8 ml of 10 mM KH_2_PO_4_ (pH 2.5). The sample was eluted from the cartridge with 4 ml acetonitrile (Sigma). The 2-methylquinoxaline (2-MQ) content was analyzed by HPLC. 2-MQ was eluted at a retention time of 7.1–7.3 min. Increase in MG was determined by calculating the ratio of peak areas between MH-S cells with and without infection. The obtained ratio was normalized with the ratio of the corresponding cell numbers.

### Cell Lysis and Immunoblot Analysis

Cells were lysed in lysis buffer containing 0.5% Nonidet P-40 (NP-40), 20 mM Tris-HCl (pH 7.6), 0.15 mM NaCl, 3 mM EDTA, 3 mM EGTA, 1 mM DTT, 2 mM sodium vanadate, and 1×proteinase inhibitor cocktail (Sigma). Lysates were cleared by centrifugation at 13,000*g* for 10 min, denatured by boiling in Laemmli buffer for 5 min, separated on 12.5% SDS-PAGE gels, and blotted onto nitrocellulose membrane. Nonspecific binding was blocked by incubating the membrane with 0.1% Tween-20/PBS containing 5% nonfat dry milk for 1 h at room temperature. Membranes were incubated in the blocking buffer with the primary antibody: caspase-3 (Cell Signaling Technology), caspase-9 (Cell Signaling Technology), or P-JNK (Sigma) overnight at 4°C, followed by incubation with horseradish peroxidases (HRP)-conjugated secondary antibody, and the specific immune complexes were detected using the Western blot enhanced chemiluminescence (ECL)-based reagent (Amersham Biosciences).

### Detection of AGE in Mycobacteria-infected Macrophages

Monoclonal antibody (mAb) against AGE (RDI, ADYGLYabm, clone 6D12) was labeled with Cy3 or Cy5 using Cy3/Cy5 Ab Labeling Kit (Amersham Biosciences) according to the manufacturer's protocol. Infected macrophages were harvested, fixed with 4% paraformaldehyde, and permeabilized in 1×PBS containing 0.1% Triton X-100. The fixed macrophages were stained with the labeled antibody and subjected to FACS analysis.

### Immunohistological Staining

Due to extensive lung disease and prevalence of MDR isolates, TB patients in Russia frequently undergo elective thoracic surgery. Destructed parts of their lungs are removed within the anatomical borders of lung segments to decrease mycobacterial load. Lung tissue was obtained at the Department of Thoracic Surgery of the Central Tuberculosis Research Institute (CTRI). At the time point of elective surgical intervention, all TB patients were under multidrug combination therapy of at least 3–4 first- and/or second-line drugs. Immediately after surgery, removed lung tissue was fixed in 4% paraformaldehyde. All procedures involving patients were reviewed and approved by the appropriate ethical review boards in both Moscow and Berlin. Informed consent was obtained from each patient in this study.

The immunohistological staining of cell surface markers was performed according to published protocols [47]. In brief, after deparaffination, tissue sections were washed with H_2_O, incubated in 1mM EDTA buffer at 2 bars for 3 min and then washed with 0.01% Tween 20 in PBS (TBS). Sections were incubated with mAb against AGE (1∶300, RDI, ADYGLYabm, clone 6D12), CD68 (1∶100; Biocarta, CM033B), pAbBCG (1∶1000; DAKO, Hamburg, Germany, B0124), or Ki-67 (1∶75; DAKO, M7240), respectively, for 1 h at room temperature. After washing in TBS, sections were incubated with goat-anti-mouse/goat-anti-rabbit polyclonal antibody labeled with alkaline phosphatase (AP; Dianova, Hamburg, Germany, 115-055-146 or 111-055-045) for 1 h. Bound antibodies were detected by color reaction using naphthol AS-bi-phosphate solution containing neo-fuchsin and sodium-nitrite (Merck, Darmstadt, Germany, 1.06549). Endogenous alkaline phosphatase activity was blocked by levamisole (Sigma, Taufkirchen, Germany, L9756). Counter staining was performed with hem-alaun (Merck).

### Measurement of TNF-α and CXCL10 Secretion

MH-S cells (2×10^6^) were treated with 0.8 mM MG in 4 ml culture medium. The growth medium was removed at different time points after MG treatment and assessed for TNF-α and CXCL10 content using Mouse TNF-alpha and CXCL10 DuoSet ELISA Development Systems, respectively, (R&D Systems) according to manufacturer's instructions. The samples from each time point were measured in triplicate. The error bars represent standard deviation from three measurements. Relative OD values were calculated by dividing each OD value with that of untreated sample (0 h).

### Gene Expression Profiling

After treatment with MG for 0, 0.5, 4.0, and 8.0 h, MH-S cells were harvested, pelleted, resuspended in Trizol (Invitrogen), and vortexed for 1 min to lyse the cells. Total RNA was extracted with chloroform and precipitated in isopropanol. The RNA pellet was washed twice with cold 70% ethanol.

The GeneChip® Mouse Genome 430 2.0 oligonucleotide microarray (Affymetrix, Santa Clara, CA, USA) comprises 45,101 probe sets, which analyze the expression level of over 39,000 transcripts and variants from over 34,000 well-characterized mouse genes. Labeling of RNA targets, hybridization, and post-hybridization procedures were performed according to protocols provided by Affymetrix. Following washing and staining, arrays were scanned twice at 1.56 µm resolution using a GeneChip® Scanner 3000 (Affymetrix, Santa Clara, CA), controlled by GCOS 1.3 software (Affymetrix). Photoemission was detected by a photomultiplier tube through a 570-nm longpass filter. Computer-generated array images were overlaid with a virtual grid, controlled by GCOS 1.3 software. This step allowed definition of each feature and alignment within known array dimensions. About 40 pixels within each feature were averaged after discarding outliers and pixels close to feature boundaries. Gene expression levels were calculated according to the average hybridization intensities of perfectly matched versus mismatched oligonucleotide probes. Arrays were scaled by GCOS 1.3 software to an average hybridization intensity of 2,500 per gene and analyzed independently. After hybridization and image analysis we excluded probe sets with absent calls in all 12 samples or with nonsignificant hybridization intensities.

The Data Mining Tool 3.1 (Affymetrix) and GeneSpring software package 7.2 (Silicon Genetics, Redwood City, CA) were used to compare the untreated control sample (0 h time point) with MG-treated samples at different time points. The data were normalized to compensate for variability in hybridizations and hybridization artefacts. The normalization consisted of three sections:

1. Data transformation: set measurements<300 to 300.
2. Normalize each chip to 50^th^ percentile of the measurements taken from that chip.
3. Normalize each gene to the median of the measurements for that gene.

The GeneSpring software package 7.2 was used to identify coexpressed genes within a set of gene expression data typically obtained using the microarray technology. The coexpression information was in turn used to help identify the biological function of the genes. To identify potential biological functions associated with gene clusters, we analyzed the Gene Ontology (GO) annotations of coexpressed genes.

Microarray data are deposited in GEO (http://www.ncbi.nih.gov/geo/; accession number: GSE3837).

### Quantitative Real-time RT-PCR of MH-S cells

MH-S cells were treated with 0.8 mM MG for 4 h with or without pretreatment with GSH. After treatment, MH-S cells were harvested, pelleted, resuspended in Trizol (Invitrogen), and vortexed for 1 min to lyse the cells. Total RNA was extracted with chloroform and precipitated in isopropanol. The RNA pellet was washed once with cold 70% ethanol. Five µg total RNA was reverse-transcribed using Reverse Transcriptase Superscript III (Invitrogen) according to the manufacturer's protocol. Quantitative real-time RT-PCR was performed using SYBR Green kit according to the manufacturer's instructions (ABI Prism 7000, Applied Biosystems) and primer pairs listed in Table S8. Relative expression levels were normalized to β-actin levels.

## Supporting Information

Figure S1 Elevated MG Levels during Mycobacterial Infection of Macrophages
(0.40 MB DOC)

Figure S2 MG Induces Apoptosis in Alveolar Macrophage Cell Line MH-S
(0.15 MB DOC)

Figure S3 MG Induces Apoptosis in the Alveolar Macrophage Cell Line MH-S
(0.17 MB DOC)

Table S1 List of genes upregulated 30 min after MG treatment with the highest fold change associated with apoptosis
(0.04 MB DOC)

Table S2 List of genes upregulated 30 min after MG treatment with the highest fold change associated with immune response
(0.04 MB DOC)

Table S3 List of genes upregulated 4 h after MG treatment with the highest fold change associated with apoptosis
(0.04 MB DOC)

Table S4 List of genes upregulated 4 h after MG treatment with the highest fold change associated with immune response
(0.04 MB DOC)

Table S5 List of genes upregulated 8 h after MG treatment with the highest fold change associated with apoptosis
(0.04 MB DOC)

Table S6 List of genes upregulated 8 h after MG treatment with the highest fold change associated with immune response
(0.04 MB DOC)

Table S7 Results of real-time RT-PCR
(0.04 MB DOC)

Table S8 Primer sequences for real-time RT-PCR
(0.04 MB DOC)

## References

[1] Kaufmann SH (2001). How can immunology contribute to the control of tuberculosis?. Nat Rev Immunol.

[2] Kaufmann SH (2000). Is the development of a new tuberculosis vaccine possible?. Nat Med.

[3] Fairbairn IP (2004). Macrophage apoptosis in host immunity to mycobacterial infections.. Biochem Soc Trans.

[4] Winau F, Weber S, Sad S, de DJ, Hoops SL, Breiden B (2006). Apoptotic vesicles crossprime CD8 T cells and protect against tuberculosis.. Immunity.

[5] Schaible UE, Winau F, Sieling PA, Fischer K, Collins HL (2003). Apoptosis facilitates antigen presentation to T lymphocytes through MHC-I and CD1 in tuberculosis.. Nat Med.

[6] Sly LM, Hingley-Wilson SM, Reiner NE, McMaster WR (2003). Survival of Mycobacterium tuberculosis in host macrophages involves resistance to apoptosis dependent upon induction of antiapoptotic Bcl-2 family member Mcl-1.. J Immunol.

[7] Mogga SJ, Mustafa T, Sviland L, Nilsen R (2002). Increased Bcl-2 and reduced Bax expression in infected macrophages in slowly progressive primary murine Mycobacterium tuberculosis infection.. Scand J Immunol.

[8] Riendeau CJ, Kornfeld H (2003). THP-1 cell apoptosis in response to Mycobacterial infection.. Infect Immun.

[9] Okado A, Kawasaki Y, Hasuike Y, Takahashi M, Teshima T (1996). Induction of apoptotic cell death by methylglyoxal and 3-deoxyglucosone in macrophage-derived cell lines.. Biochem Biophys Res Commun.

[10] Kang Y, Edwards LG, Thornalley PJ (1996). Effect of methylglyoxal on human leukaemia 60 cell growth: modification of DNA G1 growth arrest and induction of apoptosis.. Leuk Res.

[11] Du J, Suzuki H, Nagase F, Akhand AA, Yokoyama T (2000). Methylglyoxal induces apoptosis in Jurkat leukemia T cells by activating c-Jun N-terminal kinase.. J Cell Biochem.

[12] Akhand AA, Hossain K, Mitsui H, Kato M, Miyata T (2001). Glyoxal and methylglyoxal trigger distinct signals for map family kinases and caspase activation in human endothelial cells.. Free Radic Biol Med.

[13] Rodriguez-Iturbe B, Ferrebuz A, Vanegas V, Quiroz Y, Mezzano S (2005). Early and sustained inhibition of nuclear factor-kappaB prevents hypertension in spontaneously hypertensive rats.. J Pharmacol Exp Ther.

[14] Abordo EA, Thornalley PJ (1997). Synthesis and secretion of tumour necrosis factor-alpha by human monocytic THP-1 cells and chemotaxis induced by human serum albumin derivatives modified with methylglyoxal and glucose-derived advanced glycation endproducts.. Immunol Lett.

[15] Rachman H, Strong M, Ulrichs T, Grode L, Schuchhardt J (2006). Unique transcriptome signature of Mycobacterium tuberculosis in pulmonary tuberculosis.. Infect Immun.

[16] Lambrecht BN (2006). Alveolar macrophage in the driver's seat.. Immunity.

[17] Shinohara M, Thornalley PJ, Giardino I, Beisswenger P, Thorpe SR (1998). Overexpression of glyoxalase-I in bovine endothelial cells inhibits intracellular advanced glycation endproduct formation and prevents hyperglycemia-induced increases in macromolecular endocytosis.. J Clin Invest.

[18] McLellan AC, Thornalley PJ, Benn J, Sonksen PH (1994). Glyoxalase system in clinical diabetes mellitus and correlation with diabetic complications.. Clin Sci (Lond).

[19] Humeny A, Kislinger T, Becker CM, Pischetsrieder M (2002). Qualitative determination of specific protein glycation products by matrix-assisted laser desorption/ionization mass spectrometry Peptide mapping.. J Agric Food Chem.

[20] Mandl-Weber S, Haslinger B, Schalkwijk CG, Sitter T (2001). Early glycated albumin, but not advanced glycated albumin, methylglyoxal, or 3-deoxyglucosone increases the expression of PAI-1 in human peritoneal mesothelial cells.. Perit Dial Int.

[21] Thornalley PJ (1988). Modification of the glyoxalase system in human red blood cells by glucose in vitro.. Biochem J.

[22] Davidson SD, Milanesa DM, Mallouh C, Choudhury MS, Tazaki H (2002). A possible regulatory role of glyoxalase I in cell viability of human prostate cancer.. Urol Res.

[23] Chaplen FW, Fahl WE, Cameron DC (1998). Evidence of high levels of methylglyoxal in cultured Chinese hamster ovary cells.. Proc Natl Acad Sci U S A.

[24] Thornalley PJ, Jahan I, Ng R (2001). Suppression of the accumulation of triosephosphates and increased formation of methylglyoxal in human red blood cells during hyperglycaemia by thiamine in vitro.. J Biochem (Tokyo).

[25] Klingler K, Tchou-Wong KM, Brandli O, Aston C, Kim R (1997). Effects of mycobacteria on regulation of apoptosis in mononuclear phagocytes.. Infect Immun.

[26] Verheij M, Bose R, Lin XH, Yao B, Jarvis WD (1996). Requirement for ceramide-initiated SAPK/JNK signalling in stress-induced apoptosis.. Nature.

[27] Dickens M, Rogers JS, Cavanagh J, Raitano A, Xia Z (1997). A cytoplasmic inhibitor of the JNK signal transduction pathway.. Science.

[28] Johnson NL, Gardner AM, Diener KM, Lange-Carter CA, Gleavy J (1996). Signal transduction pathways regulated by mitogen-activated/extracellular response kinase kinase kinase induce cell death.. J Biol Chem.

[29] Turner NA, Xia F, Azhar G, Zhang X, Liu L (1998). Oxidative stress induces DNA fragmentation and caspase activation via the c-Jun NH2-terminal kinase pathway in H9c2 cardiac muscle cells.. J Mol Cell Cardiol.

[30] Zhang Y, Huang Y, Rishi AK, Sheikh MS, Shroot B (1999). Activation of the p38 and JNK/SAPK mitogen-activated protein kinase pathways during apoptosis is mediated by a novel retinoid.. Exp Cell Res.

[31] Lal MA, Brismar H, Eklof AC, Aperia A (2002). Role of oxidative stress in advanced glycation end product-induced mesangial cell activation.. Kidney Int.

[32] Yeh CH, Sturgis L, Haidacher J, Zhang XN, Sherwood SJ (2001). Requirement for p38 and p44/p42 mitogen-activated protein kinases in RAGE-mediated nuclear factor-kappaB transcriptional activation and cytokine secretion.. Diabetes.

[33] Abe H, Matsubara T, Iehara N, Nagai K, Takahashi T (2004). Type IV collagen is transcriptionally regulated by Smad1 under advanced glycation end product (AGE) stimulation.. J Biol Chem.

[34] Heidland A, Sebekova K, Schinzel R (2001). Advanced glycation end products and the progressive course of renal disease.. Am J Kidney Dis.

[35] Heijst JW, Niessen HW, Musters RJ, Hinsbergh VW, Hoekman K (2005). Argpyrimidine-modified Heat Shock Protein 27 in human non-small cell lung cancer: A possible mechanism for evasion of apoptosis.. Cancer Lett.

[36] Yim HS, Kang SO, Hah YC, Chock PB, Yim MB (1995). Free radicals generated during the glycation reaction of amino acids by methylglyoxal. A model study of protein-cross-linked free radicals.. J Biol Chem.

[37] Yim MB, Yim HS, Lee C, Kang SO, Chock PB (2001). Protein glycation: creation of catalytic sites for free radical generation.. Ann N Y Acad Sci.

[38] Oya T, Hattori N, Mizuno Y, Miyata S, Maeda S (1999). Methylglyoxal modification of protein. Chemical and immunochemical characterization of methylglyoxal-arginine adducts.. J Biol Chem.

[39] Du J, Suzuki H, Nagase F, Akhand AA, Ma XY (2001). Superoxide-mediated early oxidation and activation of ASK1 are important for initiating methylglyoxal-induced apoptosis process.. Free Radic Biol Med.

[40] Chiu BC, Shang X, Frait KA, Hu JS, Komuniecki E (2002). Differential effects of ageing on cytokine and chemokine responses during type-1 (mycobacterial) and type-2 (schistosomal) pulmonary granulomatous inflammation in mice.. Mech Ageing Dev.

[41] Kaufmann SH (2002). Protection against tuberculosis: cytokines, T cells, and macrophages.. Ann Rheum Dis.

[42] Kaneko H, Yamada H, Mizuno S, Udagawa T, Kazumi Y (1999). Role of tumor necrosis factor-alpha in Mycobacterium-induced granuloma formation in tumor necrosis factor-alpha-deficient mice.. Lab Invest.

[43] Law K, Weiden M, Harkin T, Tchou-Wong K, Chi C (1996). Increased release of interleukin-1 beta, interleukin-6, and tumor necrosis factor-alpha by bronchoalveolar cells lavaged from involved sites in pulmonary tuberculosis.. Am J Respir Crit Care Med.

[44] Zhang Y (2004). Persistent and dormant tubercle bacilli and latent tuberculosis.. Front Biosci.

[45] Kalapos MP (1999). Methylglyoxal in living organisms: chemistry, biochemistry, toxicology and biological implications.. Toxicol Lett.

[46] Kalapos MP (1999). On the promine/retine theory of cell division: now and then.. Biochim Biophys Acta.

[47] Ulrichs T, Lefmann M, Reich M, Morawietz L, Roth A (2005). Modified immunohistological staining allows detection of Ziehl-Neelsen-negative Mycobacterium tuberculosis organisms and their precise localization in human tissue.. J Pathol.

