# Supplementary material for: Critical Role of Methylglyoxal and AGE in Mycobacteria-Induced Macrophage Apoptosis and Activation
Source: PLoS One. 2006 Dec 20;1(1):e29. doi: 10.1371/journal.pone.0000029 (PMC1762319; doi:10.1371/journal.pone.0000029)
Supplement: Figure S1 — Elevated MG Levels during Mycobacterial Infection of Macrophages (0.40 MB DOC) [file pone.0000029.s001.doc]

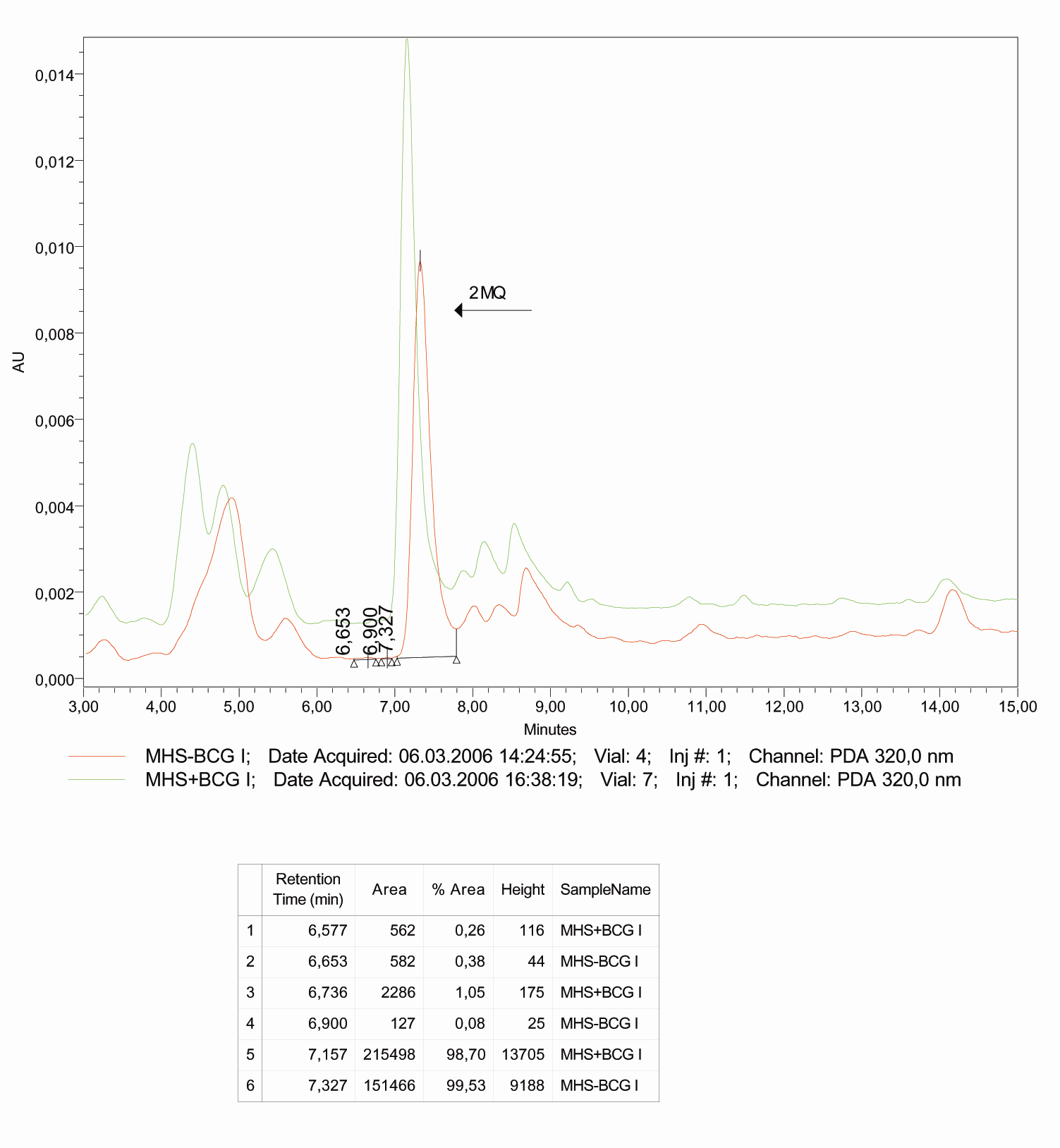


**Figure S1A**

Figures S1A–C. Elevated MG Levels during Mycobacterial Infection of Macrophages

MG was detected as 2-methylquinoxaline (2-MQ) in macrophage lysate using HPLC. MG levels are represented as the integral values of the area enclosed by the elution peak of 2-MQ in arbitrary units (AU). The curves were overlaid using a graphic processing program.


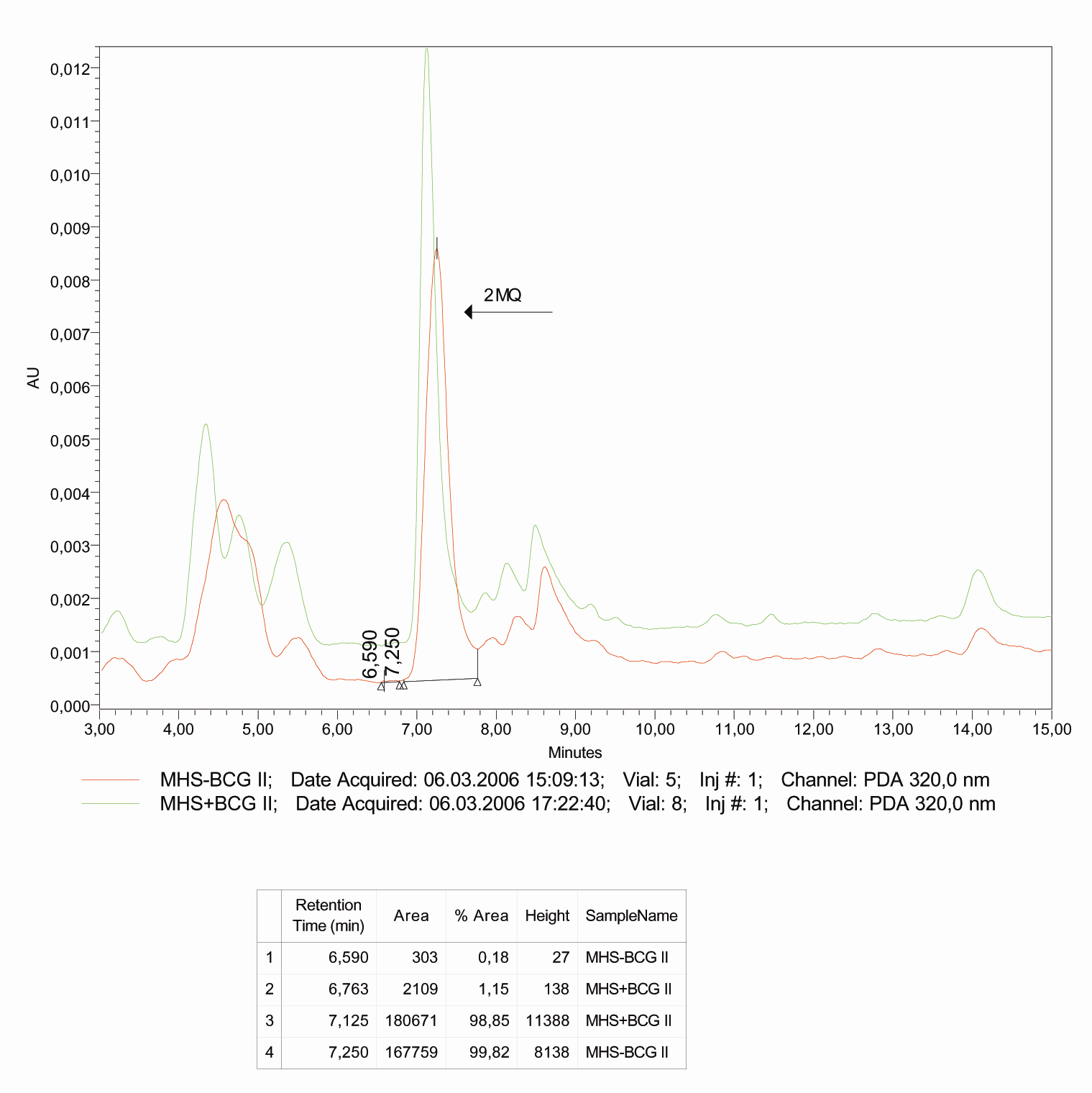


**Figure S1B**


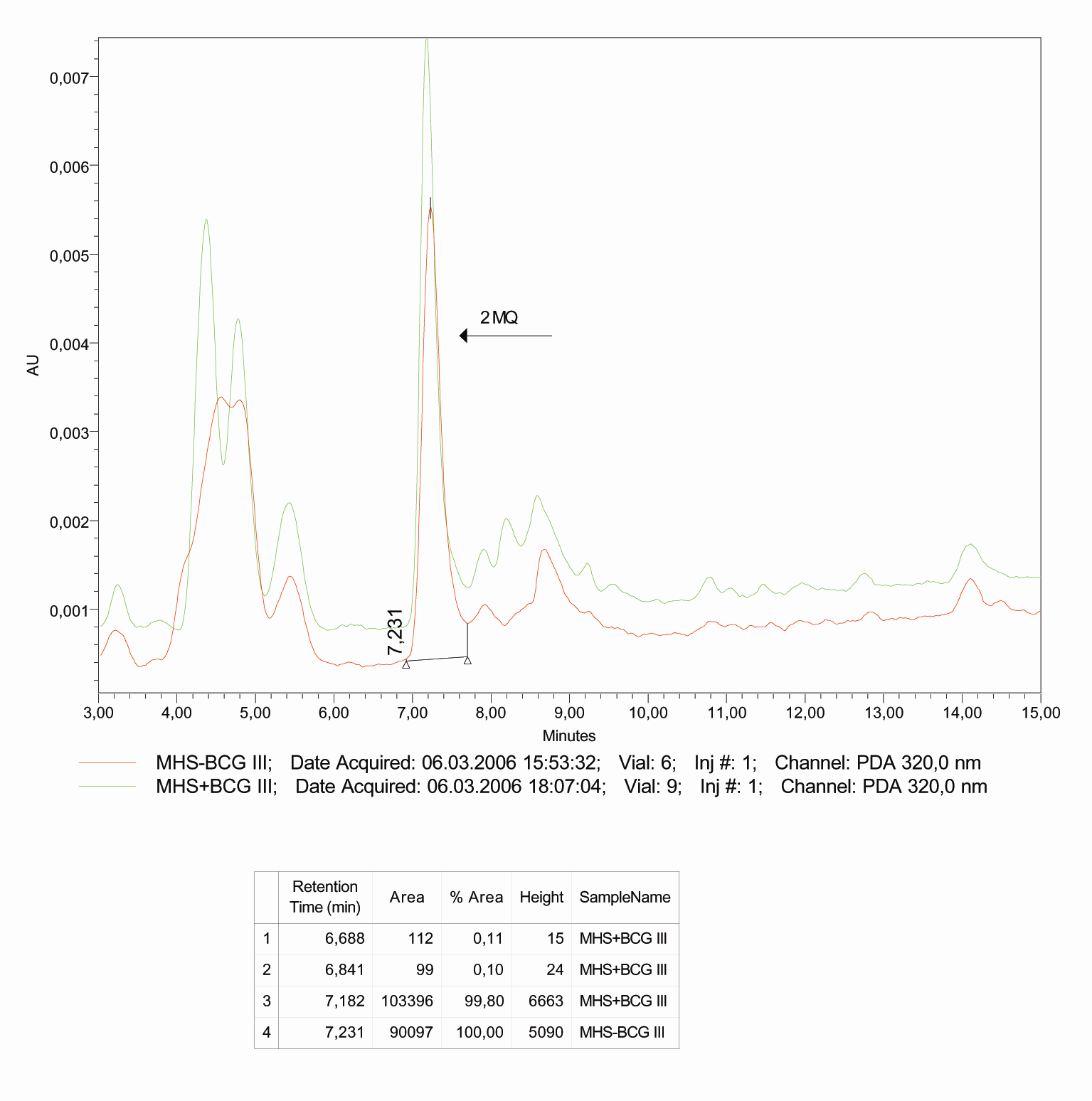
**Figure S1C**
