# Supplementary material for: Critical Role of Methylglyoxal and AGE in Mycobacteria-Induced Macrophage Apoptosis and Activation
Source: PLoS One. 2006 Dec 20;1(1):e29. doi: 10.1371/journal.pone.0000029 (PMC1762319; doi:10.1371/journal.pone.0000029)
Supplement: Figure S2 — MG Induces Apoptosis in Alveolar Macrophage Cell Line MH-S (0.15 MB DOC) [file pone.0000029.s002.doc]

**Figure S2**


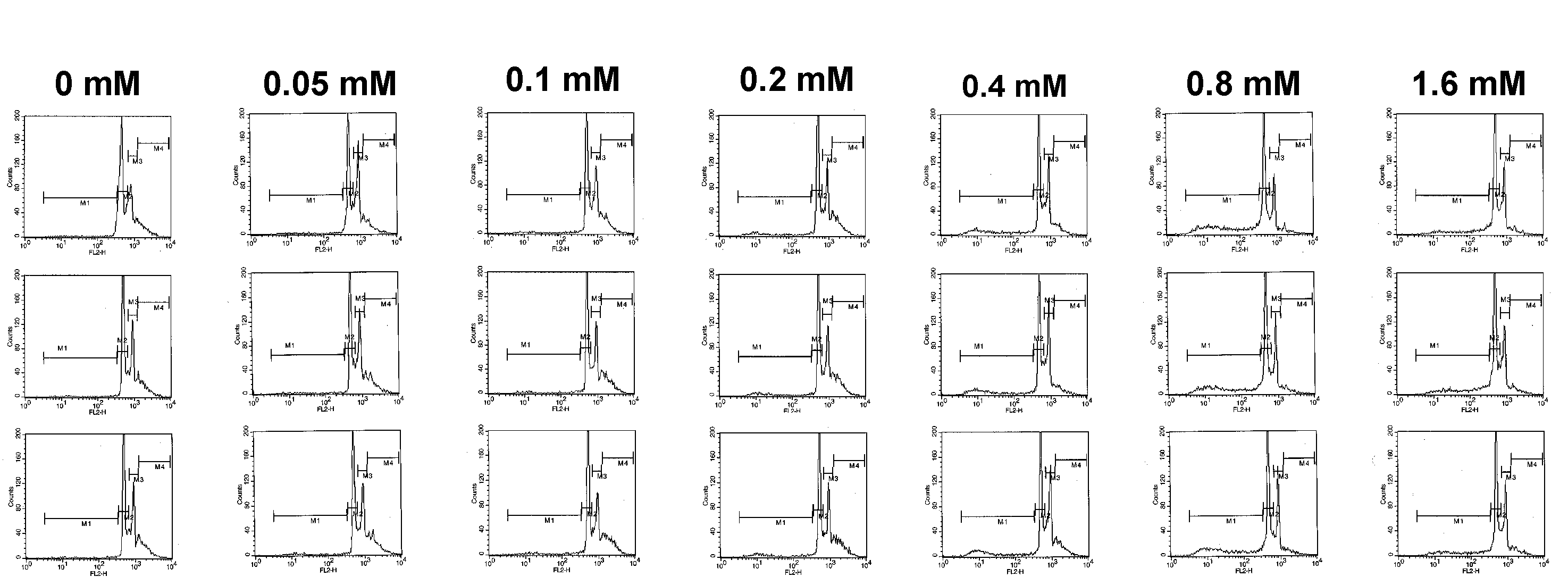


Figure S2. MG Induces Apoptosis in Alveolar Macrophage Cell Line MH-S

MH-S cells were treated with different concentrations of MG for 24 h. The cells were stained with propidium iodide and analyzed using FACS. Apoptosis was scored by the percentage of cells appearing in the area below the G1/G0 peak, which represents cells containing DNA < 2N. A representative result of FACS data from one experiment is shown here with each column representing a triplicate determination.
