# Supplementary material for: Critical Role of Methylglyoxal and AGE in Mycobacteria-Induced Macrophage Apoptosis and Activation
Source: PLoS One. 2006 Dec 20;1(1):e29. doi: 10.1371/journal.pone.0000029 (PMC1762319; doi:10.1371/journal.pone.0000029)
Supplement: Figure S3 — MG Induces Apoptosis in the Alveolar Macrophage Cell Line MH-S (0.17 MB DOC) [file pone.0000029.s003.doc]

**Figure S3**

**0 h versus 4 h**


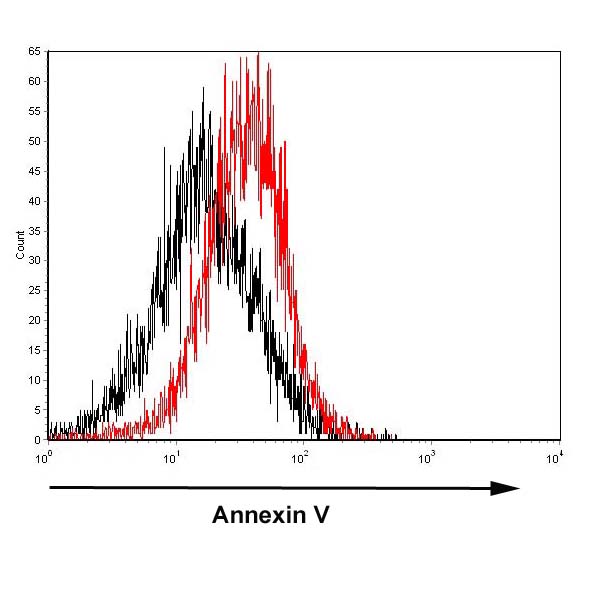


**0 h versus 6 h**


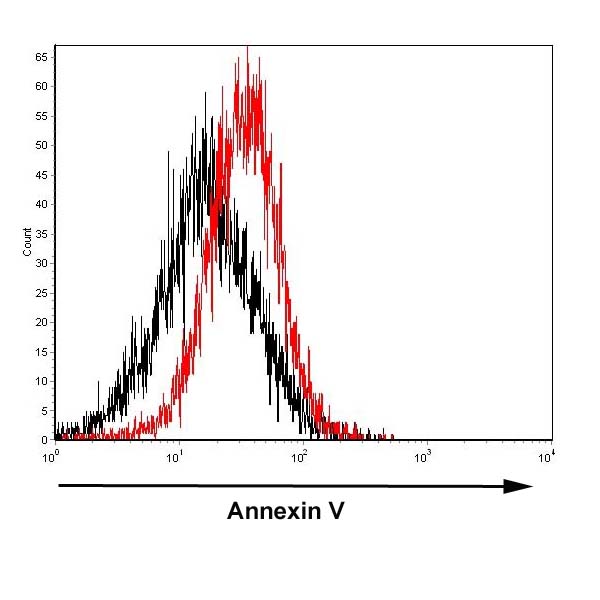


**0 h versus 24 h**


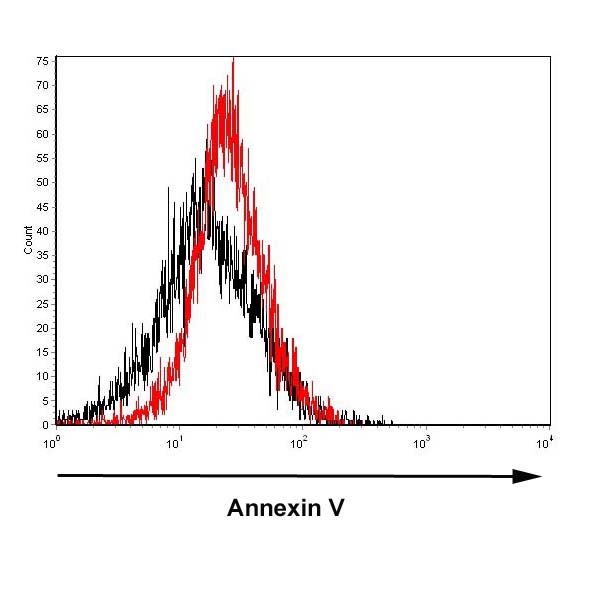


Figure S3. MG Induces Apoptosis in the Alveolar Macrophage Cell Line MH-S

MH-S cells were treated with 0.8 mM MG and harvested after MG treatment for 4, 6, or 24 h. The cells were stained with annexin V and analyzed using FACS; black line: 0 h, red line: 4, 6, or 24 h. A representative result of FACS data from one experiment is shown here with each column representing a duplicate determination.
