# Supplementary material for: Critical Role of Methylglyoxal and AGE in Mycobacteria-Induced Macrophage Apoptosis and Activation
Source: PLoS One. 2006 Dec 20;1(1):e29. doi: 10.1371/journal.pone.0000029 (PMC1762319; doi:10.1371/journal.pone.0000029)
Supplement: Table S1 — List of genes upregulated 30 min after MG treatment with the highest fold change associated with apoptosis (0.04 MB DOC) [file pone.0000029.s004.doc]

**Table S1.** List of genes upregulated 30 min after MG treatment with the highest fold change associated with apoptosis

| **Gene Name** | **Fold Change** |
| --- | --- |
| *BBP* | 5.29 |
| *AIPL1* | 4.21 |
| *TNF-α* | 3.96 |
| *DIABLO* | 2.95 |
| *COL18A1* | 2.84 |
| *DAPK1* | 2.83 |
| *DDIT3* | 2.68 |
| *CFLAR* | 2.66 |
| *RHOB* | 2.63 |
| *ELMO1* | 2.63 |
| *IGF1* | 2.48 |
| *MYC* | 2.32 |
| *GADD45B* | 2.32 |
| *1700020C11RIK* | 2.3 |
| *TNFRSF5* | 2.29 |
| *THOC1* | 2.26 |
| *TRAF1* | 2.22 |
| *WWOX* | 2.21 |
