# Supplementary material for: Critical Role of Methylglyoxal and AGE in Mycobacteria-Induced Macrophage Apoptosis and Activation
Source: PLoS One. 2006 Dec 20;1(1):e29. doi: 10.1371/journal.pone.0000029 (PMC1762319; doi:10.1371/journal.pone.0000029)
Supplement: Table S2 — List of genes upregulated 30 min after MG treatment with the highest fold change associated with immune response (0.04 MB DOC) [file pone.0000029.s005.doc]

**Table S2. List of genes upregulated 30 min after MG treatment with the highest fold change associated with immune response**

| **Gene Name** | **Fold Change** |
| --- | --- |
| *IL27RA* | 5.91 |
| *CCL12* | 5.54 |
| *CCL5* | 5.08 |
| *IL4RA* | 4.71 |
| *TNF-α* | 3.96 |
| *CCL28* | 3.88 |
| *CXCL2* | 3.69 |
| *CCL7* | 3.52 |
| *H2-DMB1* | 3.4 |
| *CCL2* | 3.04 |
| *4921509B22RIK* | 3.04 |
| *LRRC28* | 3.01 |
| *CCL4* | 2.93 |
| *IL1RN* | 2.85 |
| *CCL3* | 2.63 |
| *CXCL10* | 2.56 |
| *G1P2* | 2.47 |
| *IL24* | 2.36 |
