# Supplementary material for: Critical Role of Methylglyoxal and AGE in Mycobacteria-Induced Macrophage Apoptosis and Activation
Source: PLoS One. 2006 Dec 20;1(1):e29. doi: 10.1371/journal.pone.0000029 (PMC1762319; doi:10.1371/journal.pone.0000029)
Supplement: Table S3 — List of genes upregulated 4 h after MG treatment with the highest fold change associated with apoptosis (0.04 MB DOC) [file pone.0000029.s006.doc]

**Table S3. List of genes upregulated 4 h after MG treatment with the highest fold change associated with apoptosis**

| **Gene Name** | **Fold Change** |
| --- | --- |
| *MYC* | 12.42 |
| *AXUD1* | 11.63 |
| *DDIT3* | 10.94 |
| *TNF-α* | 9.67 |
| *OSM* | 9.51 |
| *BTG2* | 7.54 |
| *SIAH2* | 7.31 |
| *NR4A1* | 7.14 |
| *TRIB3* | 6.7 |
| *TNFRSF12A* | 6.64 |
| *RHOB* | 6.34 |
| *TRAF1* | 6.05 |
| *SIRT1* | 5.52 |
| *BBP* | 5.24 |
| *DIABLO* | 5.14 |
| *BBC3* | 4.74 |
| *CDKN1A* | 4.56 |
| *CFLAR* | 4.5 |
