# Supplementary material for: Critical Role of Methylglyoxal and AGE in Mycobacteria-Induced Macrophage Apoptosis and Activation
Source: PLoS One. 2006 Dec 20;1(1):e29. doi: 10.1371/journal.pone.0000029 (PMC1762319; doi:10.1371/journal.pone.0000029)
Supplement: Table S4 — List of genes upregulated 4 h after MG treatment with the highest fold change associated with immune response (0.04 MB DOC) [file pone.0000029.s007.doc]

**Table S4. List of genes upregulated 4 h after MG treatment with the highest fold change associated with immune response**

| **Gene Name** | **Fold Change** |
| --- | --- |
| *CXCL2* | 13.85 |
| *TNF-α* | 9.67 |
| *OSM* | 9.51 |
| *IL4RA* | 6.67 |
| *CXCL10* | 4.85 |
| *IL27RA* | 4.33 |
| *REPIN1* | 4.01 |
| *CCL28* | 3.93 |
| *RELB* | 3.72 |
| *LIF* | 3.59 |
| *IL1RN* | 3.55 |
| *TNFRSF5* | 3.54 |
| *H2-DMB1* | 3.44 |
| *CCL4* | 3.26 |
| *H2-DMB1 /// H2-DMB2* | 3.11 |
| *H2-AA* | 3.09 |
| *IFIT1* | 3.06 |
| *IL7* | 3.04 |
