# Supplementary material for: Critical Role of Methylglyoxal and AGE in Mycobacteria-Induced Macrophage Apoptosis and Activation
Source: PLoS One. 2006 Dec 20;1(1):e29. doi: 10.1371/journal.pone.0000029 (PMC1762319; doi:10.1371/journal.pone.0000029)
Supplement: Table S5 — List of genes upregulated 8 h after MG treatment with the highest fold change associated with apoptosis (0.04 MB DOC) [file pone.0000029.s008.doc]

**Table S5. List of genes upregulated 8 h after MG treatment with the highest fold change associated with apoptosis**

| **Gene Name** | **Fold Change** |
| --- | --- |
| *TRIB3* | 11.93 |
| *DDIT3* | 10.55 |
| *TRAF1* | 9.9 |
| *MYC* | 8.39 |
| *ATF5* | 8.08 |
| *ERN1* | 6.33 |
| *RABEP1* | 5.26 |
| *TNFRSF5* | 5.23 |
| *CDKN1A* | 5.1 |
| *CFLAR* | 4.86 |
| *ITGB3BP* | 4.78 |
| *DNASE2A* | 4.65 |
| *SIRT1* | 4.53 |
| *DNASE1* | 4.44 |
| *CEBPB* | 4.35 |
| *HELLS* | 4.32 |
| *SMNDC1* | 4.31 |
| *SIAH2* | 4.24 |
