# Supplementary material for: Critical Role of Methylglyoxal and AGE in Mycobacteria-Induced Macrophage Apoptosis and Activation
Source: PLoS One. 2006 Dec 20;1(1):e29. doi: 10.1371/journal.pone.0000029 (PMC1762319; doi:10.1371/journal.pone.0000029)
Supplement: Table S6 — List of genes upregulated 8 h after MG treatment with the highest fold change associated with immune response (0.04 MB DOC) [file pone.0000029.s009.doc]

**Table S6. List of genes upregulated 8 h after MG treatment with the highest fold change associated with immune response**

| **Gene Name** | **Fold Change** |
| --- | --- |
| *CXCL2* | 25.65 |
| *IL4RA* | 15.73 |
| *IL6* | 8.4 |
| *CXCL10* | 6.51 |
| *TNFRSF5* | 5.23 |
| *RELB* | 4.59 |
| *IFIT1* | 4.2 |
| *REPIN1* | 4.19 |
| *TNF-α* | 4.07 |
| *TOLLIP* | 3.95 |
| *BMI1* | 3.68 |
| *IL1RN* | 3.64 |
| *TLR2* | 3.6 |
| *G1P2* | 3.56 |
| *IL1F5* | 3.36 |
| *A430108B07RIK* | 3.34 |
| *H2-DMB1 /// H2-DMB2* | 3.26 |
| *H2-DMB1* | 3.11 |
