# Supplementary material for: Critical Role of Methylglyoxal and AGE in Mycobacteria-Induced Macrophage Apoptosis and Activation
Source: PLoS One. 2006 Dec 20;1(1):e29. doi: 10.1371/journal.pone.0000029 (PMC1762319; doi:10.1371/journal.pone.0000029)
Supplement: Table S7 — Results of real-time RT-PCR (0.04 MB DOC) [file pone.0000029.s010.doc]

**Table S7.** The results of Real-time RT-PCR

| **Gene Name** | **Fold Change** | | |
| --- | --- | --- | --- |
|  | **Microarray Data** | **Real-time RT-PCR** | |
|  |  | **MH-S + MG vs. MH-S** | **MH-S + MG vs. MH-S, + GSH** |
| TNF-α | 9.7 | 4.0 | 1.3 |
| TRAF1 | 6.0 | 17.7 | 9.0 |
| TRAF2 | 3.5 | 3.2 | 1.7 |
| CXCL10 | 4.8 | 17.4 | 4.7 |
| CXCL2 | 13.8 | 19.8 | 3.6 |
| DDIT3 | 10.9 | 21.3 | 5.5 |
| MYC | 12.4 | 57.6 | 4.0 |
| TNFRSF5 | 3.5 | 6.1 | 2.7 |
| CASP11 | 2.5 | 7.4 | 2.4 |
| TOLLIP | 2.8 | 5.0 | 1.8 |
