# Supplementary material for: Critical Role of Methylglyoxal and AGE in Mycobacteria-Induced Macrophage Apoptosis and Activation
Source: PLoS One. 2006 Dec 20;1(1):e29. doi: 10.1371/journal.pone.0000029 (PMC1762319; doi:10.1371/journal.pone.0000029)
Supplement: Table S8 — Primer sequences for real-time RT-PCR (0.04 MB DOC) [file pone.0000029.s011.doc]

**Table S8.** Primer sequences for Real-time RT-PCR

| Gene Name | **Primer sequence** | |
| --- | --- | --- |
|  | **Forward** | **Reverse** |
| β-ACTIN | TGGAATCCTGTGGCATCCATGAAAC | TAAAACGCAGCTCAGTAACAGTCCG |
| TNF-α | CCAAAGGGATGAGAAGTTCCCAAAT | CTTGGTGGTTTGTACGACGTGGG |
| TRAF1 | CTGTTCCTTCAAGGTGGTGGAATTAC | TAGCATAAAGGTGACCTGGAGAGAAGAG |
| TRAF2 | ACGTGGACCTGGAGGTACACTATGAGGT | TTCTCAGTCTCCACCATCTCTGAACAGC |
| CXCL10 | GTGGGACTCAAGGGATCCCTCTC | GTGTTGAGATCATTGCCACGATGAAA |
| CXCL2 | ACCAGGCTACAGGGGCTGTTGTG | CCTTGAGAGTGGCTATGACTTCTGTCTG |
| DDIT3 | CACCTGAAAGCAGAACCTGGTCCAC | GTTTTTGATTCTTCCTCTTCGTTTCCTG |
| MYC | AGAGCTCCTCGAGCTGTTTG | T TTCTCTTCCTCGTCGCAGAT |
| TNFRSF5 | CACTGTGAACCCAATCAAGGGCTTC | GTGGTCTCAGTGGCCATCTCCATAAC |
| CASP11 | CTTACAGCAGAGGGCATGGAGTCA | TCTCCAGAGTTCCCACCTCTGCAG |
| TOLLIP | TCTTCGATGAGCGCGCCTTCTC | AGCTGGGAGGGACGTGTAGGACAT |
